# Economic burden of disease and mortality of intracranial haemorrhage under oral FXai: a German claims data analysis

**DOI:** 10.1186/s42466-025-00366-3

**Published:** 2025-03-31

**Authors:** Hagen B. Huttner, Felix Scherg, Katarina Kopke, Michael Schultze, Nils Kossack, Stefan T. Gerner, Joji B. Kuramatsu, Stefan Schwab

**Affiliations:** 1 Department of Neurology, University Hospital Giessen, Giessen, Germany; 2 BioPharmaceuticals Medical, AstraZeneca, Hamburg, Germany; 3 ZEG – Berlin Center for Epidemiology and Health Research GmbH, Berlin, Germany; 4 WIG2 – Scientific Institute for Health Economics and Health System Research, Leipzig, Germany; 5 Department of Neurology, University Hospital Erlangen, Erlangen, Germany; 6 Department of Neurology, RoMed Klinikum, Rosenheim, Germany

**Keywords:** Anticoagulation, Intracranial haemorrhage, ICH, Oral FXai, Oral factor Xa inhibitor, Direct oral anticoagulant, DOAC

## Abstract

**Background:**

Intracranial haemorrhage (ICH) is one of the most serious complications of anticoagulant therapy with oral factor Xa inhibitors (FXai). To meet an urgent medical need of optimising treatment pathways, we assessed the frequency of ICH during oral FXai treatment, as well as the associated burden on the German healthcare system.

**Methods:**

Our study was based on a claims database comprising over 4 million people with statutory health insurance in Germany. The study included people initiating oral FXai treatment for the first time between 2016 and 2021, and who experienced ICH during a three-year treatment period. For a balanced comparison of hospitalisations, costs, and mortality, propensity score matching between patients with and without ICH was performed.

**Results:**

During the study period, 78,086 patients had started oral FXai therapy, of which 530 experienced ICH during the therapy. The incidence rate of ICH was highest within the first 90 days after the start of oral FXai therapy during follow-up with 0.64 events per 100 patient-years (PY; 95% CI: 0.52–0.77%). Three-month mortality rates were significantly higher among patients who had experienced an ICH event (39.4%; 95% CI: 35.4–43.8%), as opposed to patients without ICH (5.9%; 95% CI: 4.2–8.3%). This difference prevailed during follow-up, while mortality increased at roughly equal rates in both patient groups. Patients with ICH were on average hospitalised for 40.4 days/PY (95% CI: 35.7 days – 45.2 days) in the first year after the event; comparable patients without ICH were hospitalised for 10.8 days/PY (95% CI: 8.3 days – 13.2 days). Annual total costs per patient were €37,328 (95% CI: €32,243–€42,412) for patients with ICH, and €10,564 (95% CI: €9,298–€11,831) for patients without ICH. Hospitalisation costs were the main driver with 86.1% versus 50.8%, respectively.

**Conclusions:**

Incidence rates of ICH during oral FXai therapy were within the range of other published real-world data. Duration of hospitalisations, associated costs, and mortality were high and significantly higher for patients with ICH than for comparable patients without ICH. The high burden on the healthcare system highlights the need for preventive measures and more efficient treatment pathways for patients with ICH under oral FXai therapy.

**Supplementary Information:**

The online version contains supplementary material available at 10.1186/s42466-025-00366-3.

## Background

Anticoagulation (AC) therapy is used for the prevention of thrombotic events, such as venous thromboembolism (VTE) or stroke [6, 7] in patients with VTE or atrial fibrillation (AF) [9, 37]. Factor Xa inhibitors (FXai) are a group of AC drugs which have become increasingly more common in the last years, as they are associated with lower risk of intracranial haemorrhage (ICH) and other major bleeding events, as opposed to other AC drugs, such as vitamin K antagonists like warfarin [26]. Direct oral anticoagulants (DOAC), such as Apixaban, Edoxaban or Rivaroxaban, are oral FXai that have been well received by clinicians and patients in recent years. Still, even with the improved benefit-risk ratio, ICH remain a serious concern under DOAC therapy, as demonstrated by the surge of publications addressing DOAC-related ICH [8, 10, 15, 19, 22, 29, 36, 38].

The risk for ischemic stroke and ICH during AC therapy is generally associated with increased morbidity and mortality. For instance, a 2016 publication reported mortality rates of 28% after DOAC-associated ICH, as well as 65% of patients becoming moderately to severely disabled [30]. Even higher mortality rates after ICH were reported in the ROCKET AF trial with 48% among AF patients receiving Rivaroxaban, and annual incidence rates of 0.67 ICH events per 100 patient-years (PY) [12]. Similarly, in the ARISTOTLE trial mortality rates of 43% were reported after ICH events in AF patients receiving Apixaban [13].

With the need for AC therapy on the one hand, and the risk for ICH during AC therapy on the other hand, it is crucial to gain a better understanding of the epidemiology of ICH during AC therapy, the associated mortality, and patient characteristics, in order to improve treatment pathways. Minimising this knowledge gap may not only mitigate medical consequences but also may decrease the economic burden of these severe bleeding events on healthcare systems.

Here, we describe a real-world population of oral FXai users, assess the incidence rates of ICH and mortality, and quantify the associated health care resource utilisation (HCRU) based on a representative claims database in Germany.

## Methods

### Study design

The study presented here, was a retrospective, observational study based on claims data of the WIG2 (Scientific Institute for Health Economics and Health System Research; *Wissenschaftliches Institut für Gesundheitsökonomie und Gesundheitssystemforschung*) benchmark database. During the study period from 2016 to 2021, this database comprised pseudonymised longitudinal data of more than 4 million Germans insured in statutory health insurance and is considered representative for the German population with respect to age, gender, and morbidity [34].

### Objectives

To assess the incidence rates of ICH among patients who had initiated oral FXai, we selected patients as follows. Patients had to receive a new prescription of an oral FXai during the study period, which was also considered the index date, and they had to be between 18 and 89 years of age. The upper age limit was implemented to allow for comparisons within the framework of the international AXIOM-EUCAN study [3], and to minimise the impact of elderly patients in palliative care (e.g., in nursing homes), which would limit the informational depth of the data collected. Together with the reason for the oral FXai prescription, at least 12 months of data needed to be available prior to the index date to assess the medical history, and to ascertain that oral FXai were newly prescribed.

Patients were not considered for the study if they had used any FXai before the index date, if they were pregnant at time of oral FXai initiation, or if they had had a major bleeding event within 60 days before oral FXai initiation. Included patients were generally followed-up for up to three years. Shorter follow-up periods were possible if a patient discontinued oral FXai therapy, switched to or concomitantly used a non-FXai anticoagulant, had a major bleeding event, had a record of pregnancy, initiated palliative care, turned 90 years old in the respective calendar year, or if the patient had died. In these cases, patients were censored for analyses at the corresponding drop-out date.

Only patients with a major bleeding event during the first oral FXai treatment episode were considered for further outcomes of interest, i.e., to describe characteristics of patients who experienced an ICH event during oral FXai therapy, as well as comorbidities including the updated Charlson Comorbidity Index (CCI) [31], and comedications within one year before the event. Further outcomes of interest among these patients were the HCRU, i.e., hospitalisations and costs, as well as mortality during a follow-up period of up to three years.

### Data analysis

Population characteristics at baseline were assessed using descriptive statistics, which were stratified by age, gender, DOAC indications, comorbidities, and comedications. The proportion of patients with ICH and incidence rates of ICH were calculated including 95% confidence intervals (CI). Propensity score matching (1:1) with a caliper width of 0.1 was used to allow for a valid comparison of HCRU between patients who had experienced an ICH event during oral FXai treatment and those who had no ICH during oral FXai treatment, but who were otherwise comparable with regard to sociodemographic characteristics, comorbidities, and concomitant medication. A graph visualising the propensity score matching including the used variables is provided as supplementary material (see supplementary Figure S1) [16]. Summarised costs were differentiated by hospitalisation, outpatient, pharmacy, and indirect costs, which were then divided by the observational period. In case of incomplete follow-up periods, HCRU and costs were annualised.

As no formal hypotheses were tested in our study, no further adjustments were made (e.g., for multiple comparisons). Analyses were performed in R, version 4.2, using packages Matching, Survivak, and cmprisk.

## Results

### Study population

The cohort of incident oral FXai users comprised a total of 78,086 patients, fulfilling the inclusion criteria, and who had initiated treatment with oral FXai between January 2016 and December 2021 (Apixaban: *n* = 37,691, 48.3%; Rivaroxaban: *n* = 24,424, 31.3%; Edoxaban: *n* = 15,971, 20.5%).These patients were predominantly male (*n* = 45,256; 58.0%) with an average age of 69.9 years (± 13.4 years), and were most often prescribed FXai for AF (*n* = 56,826; 72.8%). The majority of patients had hypertension (*n* = 62,449; 80.0%), followed by gastrointestinal (*n* = 43,898; 56.2%) and urological diseases (*n* = 27,866; 35.7%). Other common comorbidities comprised heart failure (*n* = 26,444; 33.9%), type 2 diabetes (*n* = 23,334; 29.9%), chronic kidney disease (*n* = 18,422; 23.6%), and stroke (*n* = 10,428; 13.4%). A comprehensive overview of patient characteristics is provided in Table 1.

**Table 1:** Baseline characteristics and comorbidities

	Incident oral FXai users	Oral FXai users with incident ICH
N	78,086	530
Age at index date, mean (SD) [years]	69.9 (13.4)	77.4 (9.1)
Age groups, [n, %]		
< 45	4122 (5.3%)	1 (0.2%)
45–64	19,061 (24.4%)	52 (9.8%)
65–74	19,028 (24.4%)	98 (18.5%)
75–84	28,324 (36.3%)	256 (48.3%)
≥ 85	7551 (9.7%)	123 (23.2%)
Gender, [n, %]		
Male	45,256 (58.0%)	309 (58.3%)
Female	32,830 (42.0%)	221 (41.7%)
DOAC indications, [n, %]		
Venous thromboembolism	26,656 (34.1%)	122 (23.0%)
Atrial fibrillation	56,826 (72.8%)	457 (86.2%)
Non-mechanical cardiac-valve replacement	2242 (2.9%)	20 (3.8%)
Comorbidities, [n, %]		
Updated Charlson Comorbidity Index (mean, [SD])	2.4 (2.2)	3.7 (2.4)
Diabetes type 1	2324 (3.0%)	20 (3.8%)
Diabetes type 2	23,334 (29.9%)	215 (40.6%)
Chronic kidney disease	18,422 (23.6%)	208 (39.3%)
Hypercholesterolemia	21,762 (27.9%)	175 (33.0%)
Hypertension	62,449 (80.0%)	486 (91.7%)
Coronary artery disease	26,082 (33.4%)	244 (46.0%)
Stroke	10,428 (13.4%)	151 (28.5%)
- Ischemic stroke	8545 (10.9%)	123 (23.2%)
- Haemorrhagic stroke	734 (0.9%)	29 (5.5%)
- Transitory ischemic attack	3180 (4.1%)	44 (8.3%)
Heart failure	26,444 (33.9%)	263 (49.6%)
Major bleeding history > 60 days prior to FXai start	3671 (4.7%)	50 (9.43%)
Gastrointestinal diseases	43,898 (56.2%)	334 (63.0%)
Anaemia	8788 (11.3%)	93 (17.6%)
Urological diseases	27,866 (35.7%)	245 (46.2%)
Comedications, [n, %]		
History of non-FXai AC use	7339 (9.4%)	29 (5.5%)
Lipid lowering therapies	26,681 (34.2%)	266 (50.2%)
Anticancer therapies	1554 (2.0%)	17 (3.2%)
NSAIDs	26,836 (34.4%)	154 (29.1%)
Antiplatelet drugs	14,273 (18.3%)	95 (17.9%)
Antidepressants	10,341 (13.2%)	110 (20.8%)
Antivirals	1251 (1.6%)	11 (2.1%)
Corticosteroids	10,533 (13.5%)	53 (10.0%)
Gastroprotective agents	28,354 (36.3%)	252 (47.6%)
Hormone therapies	1290 (1.7%)	12 (2.3%)
Macrolides	4266 (5.5%)	20 (3.8%)

AC: anticoagulant; FXai: factor Xa inhibitors; ICH: intracranial haemorrhage; NSAID: non-steroidal anti-inflammatory drug; SD: standard deviation

### ICH under oral FXai treatment

In the study cohort of incident oral FXai users, 5,240 patients had a major bleeding event between 2016 and 2021. Among these, the first major bleeding event was an ICH in 530 patients. Nearly half of these ICH events were intracerebral haemorrhage (*n* = 249), while 158 events were subarachnoidal haemorrhage (SAH), and 184 were recorded as other sub- and epidural haemorrhage, with some patients experiencing bleeding events in multiple locations.

Compared to the overarching cohort of incident oral FXai users, this subgroup of patients who had experienced an ICH during oral FXai treatment was on average older (77.4 years, ± 9.1 years; incident oral FXai users: 69.9 years, ± 13.4 years), and had common comorbidities more often, which was also reflected in a higher average updated CCI (3.7, ± 2.4; incident oral FXai users: 2.35, ± 2.2). Patients who had experienced an ICH event were also prescribed oral FXai for AF more often (*n* = 457, 86.2%; incident oral FXai users: *n* = 56,826, 72.8%) (Table 1).

**Fig. 1:**
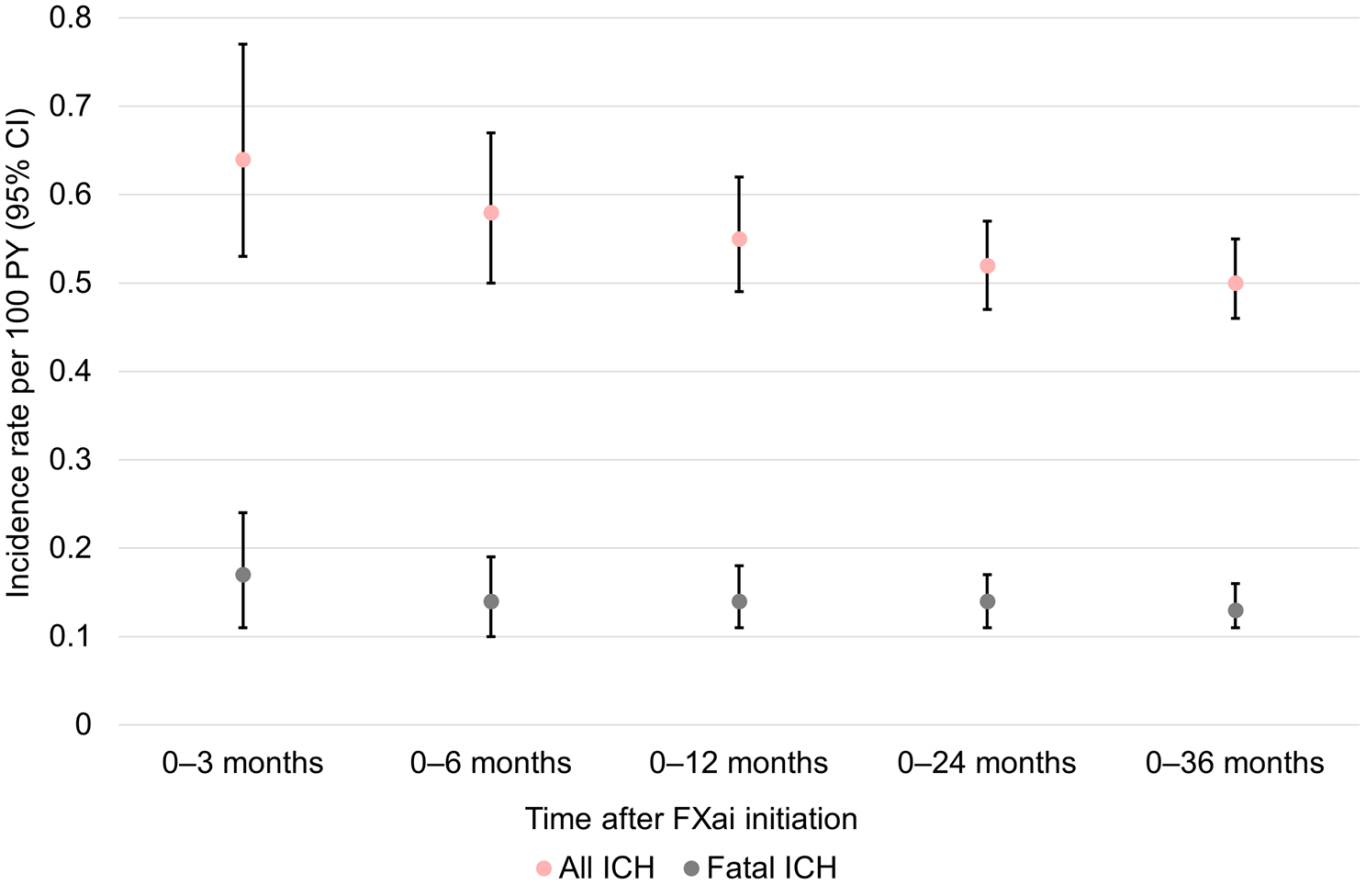
Cumulative incidence rates of ICH events per 100 patient-years [PY] over three years after initiation of treatment with oral FXai. Graph based on 471 ICH events and 125 fatal ICH events over a three-year period. Incidence rates were defined as the total number of incident bleeding events divided by the total person time at risk. CI: confidence interval; FXai: factor Xa inhibitors; ICH: intracranial haemorrhage; PY: patient-years

The highest cumulative incidence rates of ICH were observed during the first three months after oral FXai initiation with 0.64 events per 100 PY (95% CI: 0.52–0.77). Cumulative incidence rates were lower over a three-year observational period with 0.50 events per 100 PY (95% CI: 0.45–0.54). Similarly, the incidence rate of fatal ICH was highest during the first three months after oral FXai initiation with 0.17/100 PY (95% CI: 0.11–0.24), as opposed to the three-year fatal incidence rate of 0.13/100 PY (95% CI: 0.11–0.16) (Fig. 1). With respect to bleeding types, the highest incidence rates were observed for intracerebral haemorrhage during the first three months after the event (0.30 events per 100 PY; 95% CI: 0.23–0.40), which was also associated with the highest mortality (0.09 fatal events per 100 PY; 95% CI: 0.05–0.15). An overview of incidence rates of other intracranial bleeding types is provided as supplementary Fig. 2 [17].

### Mortality after bleeding event

**Fig. 2:**
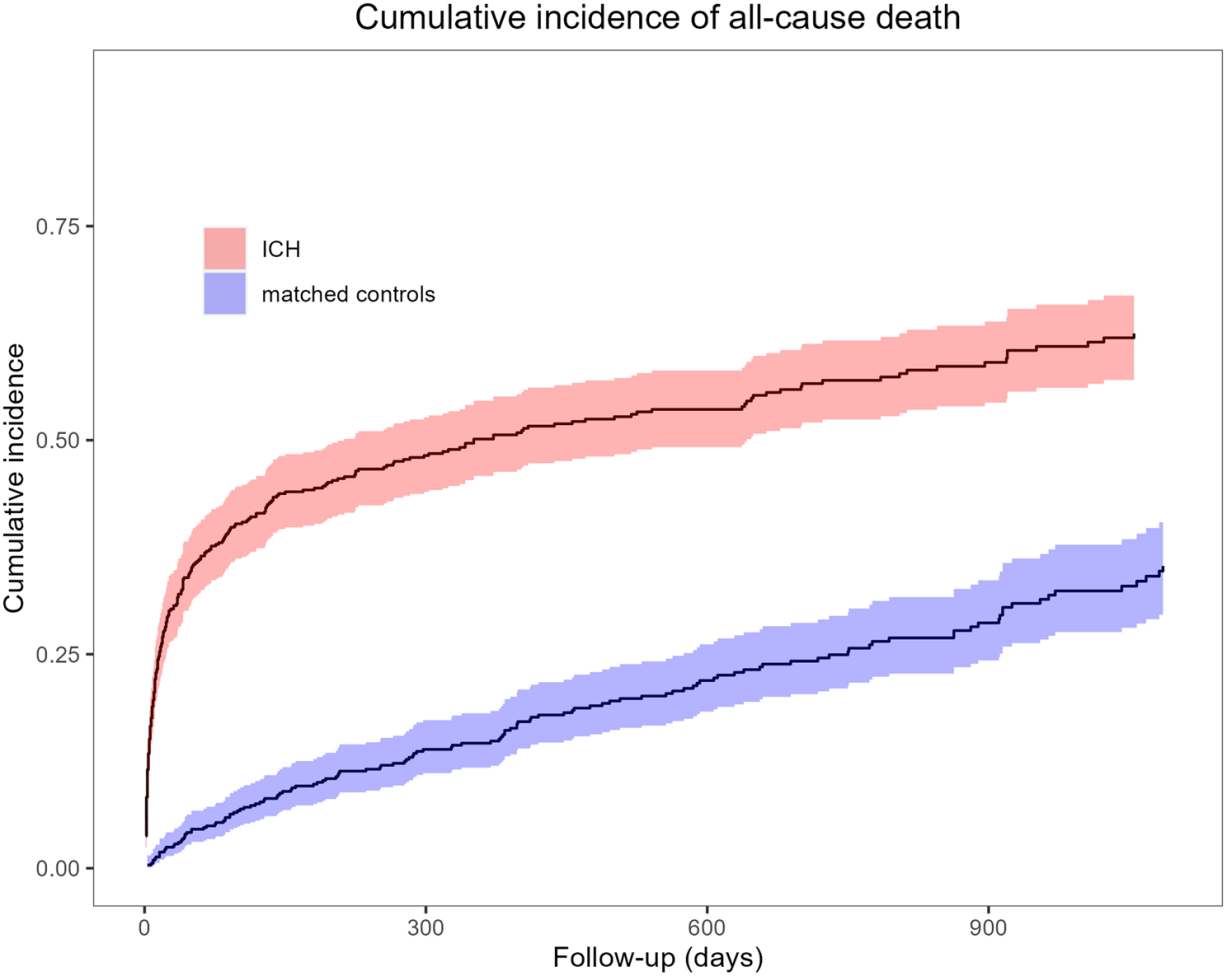
Cumulative incidence of (all-cause) death in patients who had experienced an ICH event compared to matched patients who had not experienced an ICH event. ICH: intracranial haemorrhage

Of 530 patients who had experienced an ICH event, 143 patients (27.0%) did not survive this first bleeding event. With 39.4% (95% CI: 35.4–43.8%), three-month mortality rates were significantly higher among patients who had experienced an ICH event, as opposed to patients without an ICH event (5.9%; 95% CI: 4.2–8.3%). This difference prevailed over the three-year observational period, while mortality rates increased over the three-year follow-up period at roughly equal rates in both patient groups. Of the 143 deaths of the first bleeding event, 81 patients (56.6%) had intracerebral haemorrhage, 41 patients (28.7%) had SAH, and 44 patients (30.8%) had other intracranial bleeding. Figure 2 provides a comprehensive overview of mortality rates between matched patient groups.

### HCRU after bleeding event

The 530 patients who had experienced an ICH were hospitalised for 40.4 days per PY (95% CI: 35.7 days – 45.2 days) on average during the first year after the bleeding event. During this time, patients caused average costs of €37,328 per PY (95% CI: €32,243–€42,412), which were mainly driven by hospitalisation costs (86.1%; €32,125; 95% CI: €27,186–€37,063). Other cost factors included outpatient visits, pharmacy costs, and indirect costs (i.e., absence of work). Over the three-year observational period, average hospitalisation increased to 62.8 days per patient (95% CI: 53.6 days – 72.0 days). Costs accumulated to an average of €60,358 per patient (95% CI: €50,116–€70,601) over three years, which remained mostly attributable to hospitalisations (79.5.4%; €47,999; 95% CI: €38,178–€57,819).

In contrast, matched patients with comparable demographic and medical baseline characteristics who had not experienced an ICH during oral FXai treatment, were on average hospitalised for 10.8 days per PY (95% CI: 8.3 days – 13.2 days) over a 12-month period, i.e., about 30 days fewer than patients who had experienced an ICH. Similarly, average costs over 12 months were about €27,000 lower (€10,564 per PY; 95% CI: €9,298–€11,831) among patients who had not experienced an ICH as opposed to those who had had an ICH event. Total costs were again driven by hospitalisations, albeit to a lesser degree than observed among patients who had experienced an ICH event (50.8%; €5,370 per PY; 95% CI: €4,586–€6,154). A comprehensive overview of HCRU between matched patient groups is provided in Table 2.

**Table 2:** All-cause HCRU (hospitalisation and costs)

	1 year since index date		2 years since index date		3 years since index date	
	ICH	Matched control	ICH	Matched control	ICH	Matched control
All-cause HCRU; mean duration per PY (95% CI)						
Length of hospital stays [days]	40.4 (35.7–45.2)	10.8 (8.3–13.2)	51.1 (44.2–58.0)	20.0 (14.3–25.8)	62.8 (53.6–72.0)	28.6 (18.9–38.3)
Work absences [days]	12.1 (7.3–17.0)	4.3 (1.7–6.9)	17.1 (10.0–24.1)	6.6 (2.7–10.4)	21.7 (12.8–30.6)	8.9 (4.0–13.8)
Cumulative costs; mean costs per PY (95% CI)						
Outpatient [€]						
GP visits	604 (551–657)	582 (533–632)	1057 (968–1146)	1020 (942–1099)	1473 (1349–1597)	1456 (1348–1565)
Specialist visits	568 (490–647)	686 (564–807)	952 (833–1072)	1349 (1087–1612)	1484 (1301–1667)	1990 (1571–2409)
Inpatient [€]						
Hospitalisations (> 24 h)	32,125 (27186–37063)	5370 (4586–6154)	39,342 (32035–46649)	9087 (7858–10316)	47,999 (38178–57819)	12,735 (11117–14352)
Pharmacy [€]						
Prescriptions	2537 (2130–2944)	3395 (2586–4204)	4748 (3873–5624)	6277 (4854–7701)	6733 (5513–7953)	8914 (6826–11003)
Indirect costs [€]						
Cost of absence from work	1493 (894–2092)	531 (215–848)	2098 (1233–2963)	807 (333–1282)	2670 (1579–3762)	1093 (486–1699)
**Total overall cost [€]**	**37,328 (32243–42412)**	**10,564 (9298–11831)**	**48,197 (40607–55788)**	**18,541 (16316–20767)**	**60,358 (50116–70601)**	**26,188 (22978–29398)**

CI: confidence interval; GP: general practitioner; HCRU: health care resource utilisation; ICH: intracranial haemorrhage; PY: patient-year

## Discussion

### General interpretation

While oral FXai are an effective group of drugs that have demonstrated benefits over previous AC therapies, ICH remain one of the most serious concerns. Here, we provide valuable insight in the real-world occurrence of ICH in patients newly started on oral FXai treatment.

The study population mostly comprised elderly, predominantly male patients with arterial hypertension, gastrointestinal diseases, or heart failure as most common comorbidities. This aligns with the fact that most patients had received oral FXai for the treatment of AF, which in turn is often associated with comorbidities such as hypertension or heart failure [2, 32].

With an observed incidence rate of 0.64/100 PY during the first three months after FXai initiation, and 0.55/100 PY over a 12-month period, respectively, our findings are at the lower end of published evidence. While 12-month incidence rates of ICH as low as 0.4/100 PY [1] and 0.5/100 PY [28] have been reported during FXai therapy, other publications mention 12-month incidence rates of 0.56/100 PY for the United Kingdom and 0.72/100 PY for Canada [3], and a recent publication from Spain even reported a 12-month incidence rate of 0.9/100 PY [4]. However, differences between healthcare systems, data sources and treatment pathways may limit a direct comparison between published values.

Still, the risk for ICH appeared to be highest early after FXai initiation, as indicated by the highest observed incidence rates within three months, compared to a lower cumulative incidence rate over the entire follow-up period. Generally, we assume that our reported incidence rates of ICH may slightly underestimate the occurrence of ICH, as only the first bleeding event was recorded for our analysis. Therefore, patients could have been excluded due to minor bleeding events before the ICH, as multiple or subsequent bleeding events were not considered, which may not necessarily have occurred during FXai therapy any longer. Irrespective of a potential underestimation of overall incidence rates, the relative proportions by bleeding types (intracerebral, subarachnoidal, or other haemorrhage) can be assumed to be reflective of real-world occurrence due to corresponding ICD-10 codes. Intracerebral haemorrhage was the most common bleeding type, which was also associated with higher mortality rates than other bleeding types, which may be attributed to the more limited treatment options. While extracerebral bleeding events may allow direct haemostatic interventions, this is often not possible in acute intracerebral haemorrhage. Hence, conservative treatments are the essential options including blood pressure management and reversal of oral anticoagulation, often focused within a care bundle to minimise further haematoma enlargement [14, 35, 39].

The matched analyses of HCRU between patients who had experienced an ICH event and those who had not, showed noteworthy differences in costs and mortality. While major bleeding events are associated with increased HCRU, costs, mortality, and overall burden on the healthcare system, our findings quantify this difference to comparable patients without major bleeding events, and thus highlight the importance of preventive measures and pronounces strongly the need for optimal acute and long-term treatment to ameliorate ICH-associated sequelae [21]. Even more so, ICH appears to be associated with a higher HCRU, costs, and mortality, than other bleeding types, such as e.g., gastrointestinal bleeds [5, 24].

Our observed average costs of €37,328 per PY within the first year after an ICH event were considerably higher than similar studies from other European countries had reported. For instance, a claims data analysis from Denmark reported total average costs over a three-year period of around €18,000 per patient among AF patients who had experienced ICH, which was almost exclusively driven by hospitalisation costs [18]. Closer to our observations, a Finnish study focusing on intracerebral haemorrhage reported average costs of €49,754 per patient in the first year after the event [33]. However, this estimate also included costs for rehabilitation, tertiary hospital stays, and social security costs. Thus, with €21,744, the initial hospitalisation costs were still lower than our estimates of €32,125 per PY for a comparable time period. Admittedly, the generalisability of such simplified cost comparisons may be limited by differences in healthcare systems and reimbursement modalities. Nevertheless, it becomes evident that more cost-efficient treatment pathways are principally available in comparison to the exceptionally high HCRU and costs as observed in our study. Even though particularly costly cases may have increased our estimates, there is a need for more time- and cost-effective treatment pathways to lessen the economic burden of FXai-associated ICH. For instance, treatment protocols focusing on an early intervention and streamlining the emergency treatment of intracerebral haemorrhage, may increase the likelihood of a faster recovery overall [11, 20, 21, 23]. In addition, the implementation of a hyperacute care bundle, comprising anticoagulation reversal, intensive blood pressure lowering, neurosurgery, and access to critical care, led to significantly decreasing 30-day case fatality rates in England and Wales [27]. Similarly, a recent investigation by Mrochen and colleagues observed significantly reduced mortality after intracerebral haemorrhage when applying a bundled care treatment, focusing on a reduction of blood pressure, glycaemic control, and maintenance of normothermia [25]. These findings were in line with those of the INTERACT-3 study [23], which employed a similar care bundle in patients with acute cerebral haemorrhage that additionally comprised the rapid reversal of warfarin-related anticoagulation. The study found that the implementation of the care bundle led to improved functional outcomes as indicated by significant changes in the modified Rankin scale ratings among patients who had received the acute bundled care treatment. In light of these findings, it stands to reason that improved functional outcomes after implementation of acute bundled care are therefore also associated with a general reduction in HCRU.

### Strengths and limitations

Like any study, our study also has limitations, which are mostly inherent with the nature of claims data. For one, claims data is recorded for reimbursement purposes rather than scientific research. With this, causal relationships can only be inferred but usually not be verified.

For example, informative clinical characteristics about the severity of bleeding events, such as e.g., the bleeding volume, are not reported in claims data, which limits the interpretation of hospitalisation duration and costs. In addition, fatal ICH events outside of hospitals were not captured as cause of death is not routinely reported in German claims data. Therefore, mortality rates may also be slightly underestimated. Further, it would have been valuable to see a comparison of ICH-patients under oral anticoagulation *versus* those without. However, the available data sources consisted only of registry data of patients with fXa-intake, why we were only able to compare – among patients with intake of fXa-inhibitors – those who developed an ICH *versus* those who did not. In addition, the study period (2016–2021) included only a short duration after Ondexxya’s approval in Germany which restricts our ability to conduct a comprehensive analysis of its impact on treatment costs. Moreover, the complexity of prothrombin complex concentrate (PCC) dosing, which is based on patient weight, and the lack of data on individual patient weights and specific PCC dosages used further complicates direct cost comparisons. Finally, the dataset requested did only focus on fXa-inhibitors why the available dataset did not contain on thrombin inhibitors like Dabigatran.

On the other hand, our study was based on a very large nationally representative database [34], which provided a robust foundation for real-world observations. Considering the limitations mentioned above, we believe that the reported estimates are nevertheless a reasonable approximation of the real-world occurrence of ICH under oral FXai therapy and associated outcomes. The choice of rather strict inclusion and exclusion criteria ensured that only patients with a new prescription of oral FXai were included, who had no prior history of major bleeding events. Therefore, we believe that our results rather err on the conservative side.

Furthermore, patients’ immobility and the chance of living in nursing homes increases with age. This may have resulted in potential incomplete recording of routine care data, such as comorbidities, symptoms, referrals to specialists, lab tests, and potential confounding factors including information on living in nursing homes. Hence, the age restriction of 90 years was intended to select a study cohort with more complete information and to facilitate the interpretation of study findings.

## Conclusions

Our study provides valuable insights in the real-world occurrence of ICH events during treatment with oral FXai. While we generally observed lower incidence rates than reported elsewhere, HCRU and costs appeared exceptionally high. By quantifying the difference in HCRU and costs between patients with and without major bleeding events, our findings emphasise the importance of standardised practices in acute care, rapid intervention, and implementation of care bundles to improve both short- and long-term outcomes for patients, which may ultimately also mitigate economic consequences of FXai-associated ICH.

## Electronic supplementary material

Below is the link to the electronic supplementary material.

Supplementary Material 1

Supplementary Material 2

## Data Availability

The datasets generated and analysed in this study are not publicly available due to data protection laws. Raw data are not publicly available to preserve individuals’ privacy under the European General Data Protection Regulation. Aggregated data may be made available upon reasonable request.

## Abbreviations

AC: Anticoagulation
AF: Atrial fibrillation
CCI: (updated) Charlson Comorbidity Index
CI: Confidence interval
DOAC: Direct oral anticoagulants
FXai: Factor Xa inhibitors
GP: General practitioner
HCRU: Health care resource utilisation
ICH: Intracranial haemorrhage
NSAID: Non-steroidal anti-inflammatory drug
PCC: Prothrombin Complex Concentrate
PY: Patient-years
SAH: Subarachnoidal haemorrhage
SD: Standard deviation
VTE: Venous thromboembolism
WIG2: Scientific Institute for Health Economics and Health System Research (Wissenschaftliches Institut für Gesundheitsökonomie und Gesundheitssystemforschung)
