# Supplementary figures and images for: Economic burden of disease and mortality of intracranial haemorrhage under oral FXai: a German claims data analysis
Source: Neurol Res Pract. 2025 Mar 31;7(1):21. doi: 10.1186/s42466-025-00366-3 (PMC11956223; doi:10.1186/s42466-025-00366-3)

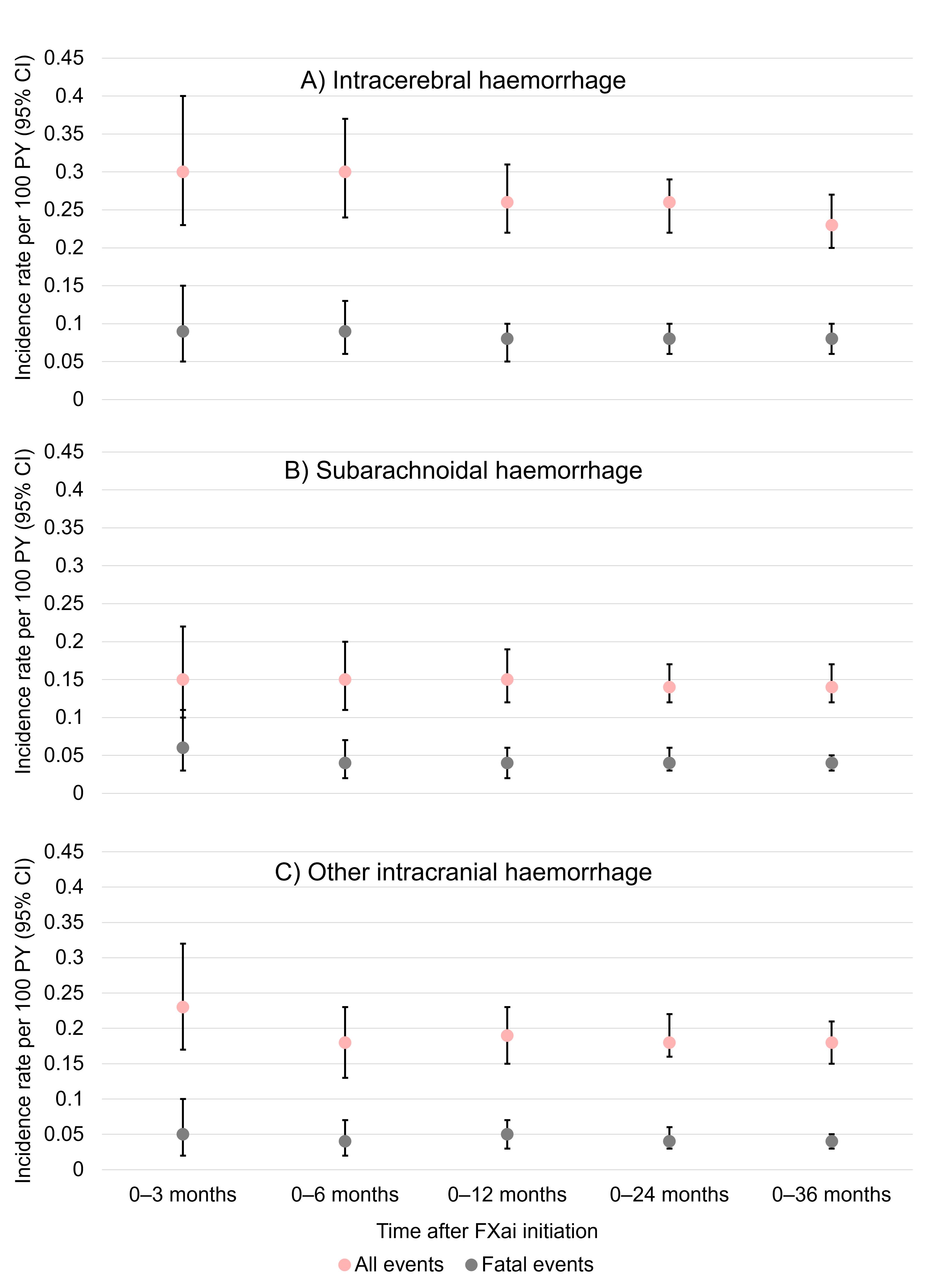

Supplement: Supplementary file 1 — Supplementary Material 1 [file 42466_2025_366_MOESM1_ESM.png]

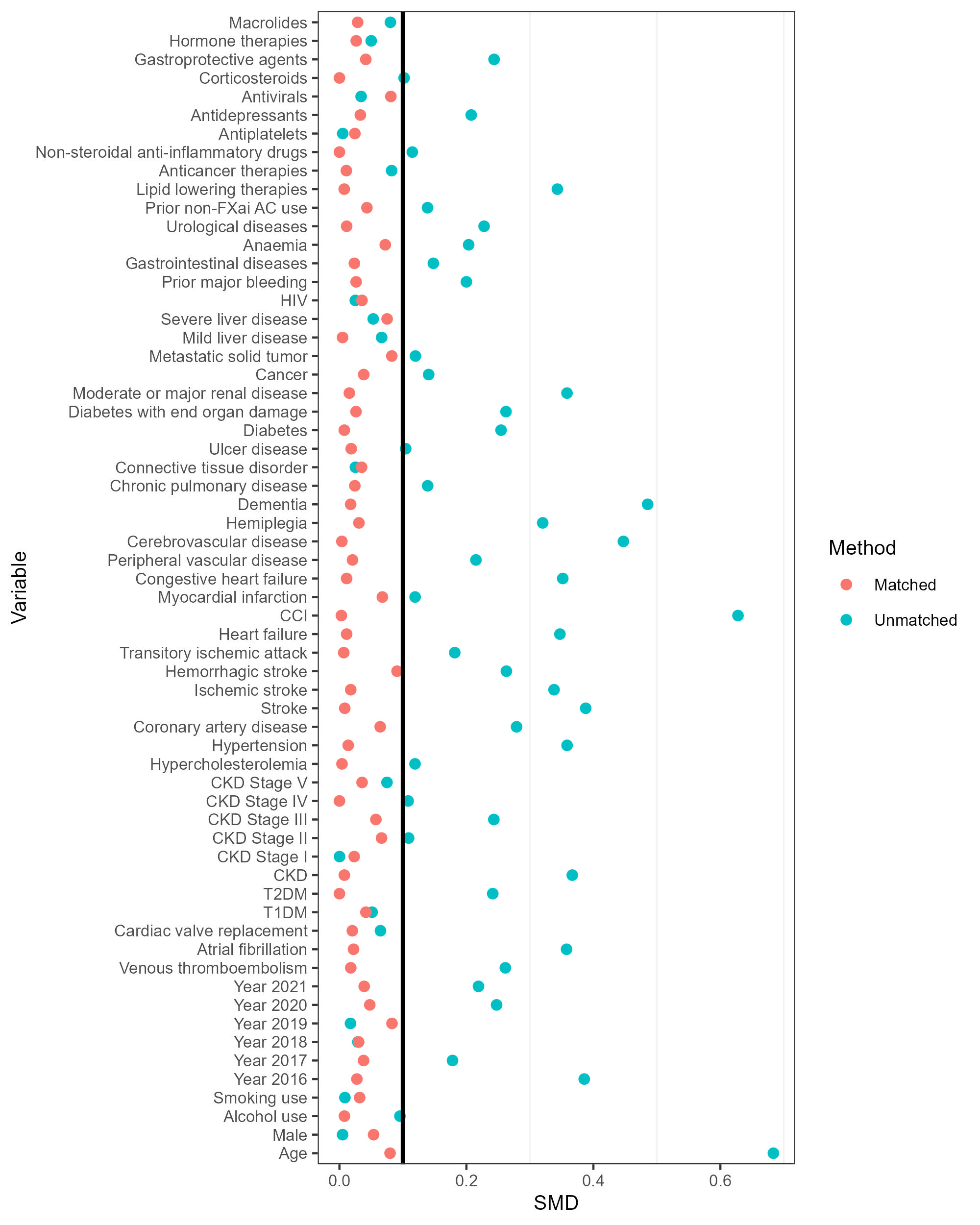

Supplement: Supplementary file 2 — Supplementary Material 2 [file 42466_2025_366_MOESM2_ESM.png]
